# Changes in cerebral autoregulation, stroke‐related blood biomarkers, and autonomic regulation after patent foramen ovale closure in severe migraine patients

**DOI:** 10.1111/cns.14244

**Published:** 2023-05-08

**Authors:** Zhen‐Ni Guo, Yang Qu, Yongsheng Gao, Yingqi Xing, Hongyin Ma, Jia Liu, Yu‐Zhu Guo, Junlei Chang, Peng Zhang, Hang Jin, Xin Sun, Ke Han, Han‐Hwa Hu, Qianyan He, David Martin Simpson, Yi Yang

**Affiliations:** ^1^ Stroke Center, Department of Neurology The First Hospital of Jilin University Changchun China; ^2^ Neuroscience Research Centre The First Hospital of Jilin University Changchun China; ^3^ Department of Cardiac Surgery The First Hospital of Jilin University Changchun China; ^4^ Center for Neurovascular Ultrasound, Department of Neurology The First Hospital of Jilin University Changchun China; ^5^ Laboratory for Engineering and Scientific Computing, Institute of Advanced Computing and Digital Engineering, Shenzhen Institute of Advanced Technology Chinese Academy of Sciences Shenzhen China; ^6^ Center for Protein and Cell‐Based Drugs Institute of Biomedicine and Biotechnology, Shenzhen Institute of Advanced Technology, Chinese Academy of Sciences Shenzhen China; ^7^ Cerebrovascular Disease Research Center, Department of Neurology, Seventh Affiliated Hospital Sun Yat‐sen University Shenzhen China; ^8^ Cerebrovascular Treatment and Research Center, College of Medicine Taipei Medical University Taipei Taiwan; ^9^ Institute of Sound and Vibration Research University of Southampton Southampton UK

**Keywords:** autonomic regulation, dynamic cerebral autoregulation, migraine, patent foramen ovale, stroke

## Abstract

**Aims:**

This study aimed to investigate changes in dynamic cerebral autoregulation (dCA), 20 stroke‐related blood biomarkers, and autonomic regulation after patent foramen ovale (PFO) closure in severe migraine patients.

**Methods:**

Patent foramen ovale severe migraine patients, matched non‐PFO severe migraine patients, and healthy controls were included. dCA and autonomic regulation were evaluated in each participant at baseline, and within 48‐h and 30 days after closure in PFO migraineurs. A panel of stroke‐related blood biomarkers was detected pre‐surgically in arterial‐and venous blood, and post‐surgically in the arterial blood in PFO migraineurs.

**Results:**

Forty‐five PFO severe migraine patients, 50 non‐PFO severe migraine patients, and 50 controls were enrolled. The baseline dCA function of PFO migraineurs was significantly lower than that of non‐PFO migraineurs and controls but was rapidly improved with PFO closure, remaining stable at 1‐month follow‐up. Arterial blood platelet‐derived growth factor‐BB (PDGF‐BB) levels were higher in PFO migraineurs than in controls, which was immediately and significantly reduced after closure. No differences in autonomic regulation were observed among the three groups.

**Conclusion:**

Patent foramen ovale closure can improve dCA and alter elevated arterial PDGF‐BB levels in migraine patients with PFO, both of which may be related to the preventive effect of PFO closure on stroke occurrence/recurrence.

## Graphical abstract

Patent foramen ovale (PFO) closure can improve dynamic cerebral autoregulation (dCA) and alter elevated arterial blood platelet‐derived growth factor‐BB (PDGF‐BB) levels in migraine patients with PFO, both of which may be related to the preventive effect of PFO closure on stroke occurrence and recurrence in these patients.

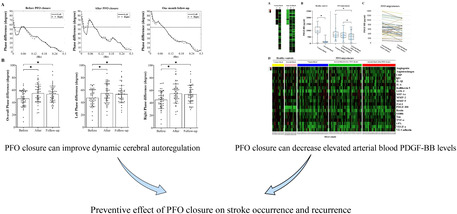

## 1 INTRODUCTION

Previous studies have reported that ischemic stroke is more common in patients with migraine, with 5.4% and 0.7% in migraineurs and controls, respectively,^1^, ^2^ the mechanisms are largely unknown. Several recent studies have found a strong association among migraine, stroke, and patent foramen ovale (PFO),^3^, ^4^ and thus PFO may be the link between migraine and stroke. It is important to explore whether the presence of PFO in the migraine population causes specific pathophysiological changes that predispose migraine patients to stroke.

The brain is highly dependent on constant and stable cerebral blood flow (CBF) despite changes in cerebral perfusion pressure and arterial blood pressure (ABP). Dynamic cerebral autoregulation (dCA) is critical in regulating cerebral hemodynamics and its dysfunction plays a role in the occurrence and progression of stroke,^5^ as an impaired dCA may increase an individual's susceptibility to stroke and impair the cerebrovascular function of clearing away the microemboli.^6^ Interestingly, a previous study found that dCA is impaired in migraine patients with PFO, especially in those with a large right‐to‐left shunt.^7^ Thus, dCA impairment may be an intermediate link between PFO and stroke in a migraine patient. Accordingly, whether the PFO closure can alleviate impaired dCA is a key concern and is still unclear.

In patients with a PFO, venous blood can enter the arterial blood circulation through the oval foramen, which may produce alterations in several stroke‐related blood biomarkers in the arterial blood, such as in homocysteine,^8^ related to the increased risk of stroke occurrence. Identifying the changes in stroke‐related blood biomarkers in migraine patients with PFO before and after PFO closure may help us understand the mechanisms of PFO‐associated stroke and pinpoint the targets for its management.

Traditional risk factors for ischemic stroke include body stressors that may lead to autonomic dysfunction, which can be reflected by beat‐to‐beat heart rate variability (HRV).^9^ Previous studies have reported that PFO is closely related to autonomic dysfunctional disorders such as irritable bowel syndrome.^10^ Thus, it is worth exploring the characteristics of autonomic function in migraine patients with PFO and the effect of PFO closure on autonomic functioning. In the present study, we investigated whether PFO closure affects dCA function, blood biomarkers, and autonomic function in severe migraine patients.

## 2 METHODS

The raw data of this study are available from the corresponding author upon reasonable request. This prospective study was approved by the ethics committee of the First Hospital of Jilin University (2012–111). Written informed consent was obtained from all participants. The participants had the right to withdraw at any time point during the course of the study.

### 2.1 Participants

Severe migraine was defined as Headache Impact Test‐6 score > 55.^11^ Severe migraine patients (aged 18–65 years) with a large right‐to‐left shunt (contrast‐enhanced transcranial Doppler >10 microbubbles)^12^ were screened from the Department of Neurology, and who were scheduled to undergo PFO closure at the Department of Cardiac Surgery from January 2013 to June 2018 were included as PFO migraineurs group in this study. The effect of PFO closure surgery was determined by consultation with the cardiac surgeons, neurologists, cardiologists, radiologists, and sonographers taking into consideration the patient's wishes.

The exclusion criteria include: (1) insufficient bilateral temporal bone window for insonation of the middle cerebral artery (MCA); (2) extracranial/intracranial artery stenosis (including mild, moderate, and severe stenosis) or occlusion^13^ [measured with an EMS‐9 PB transcranial Doppler detector (Delica) and an iU22 ultrasound (Phillips)]; (3) patients who had brain tumor, stroke, white matter lesions, and other structural abnormities of brain screened with brain CT scan, myocardial infarction, and unstable angina within the past 6 months, or atrial fibrillation; (4) who were administered medications known to affect vasomotion within 1 week before the study; (5) who had large residual shunting measured with contrast‐enhanced transcranial Doppler (>10 microbubbles) at the 1‐month follow up; (6) who did not agree to participate in the study.

Age‐ and sex‐matched severe migraineurs without a PFO (determined by contrast‐enhanced transcranial Doppler) and healthy migraine‐free volunteers were also enrolled as non‐PFO migraineurs and healthy controls. Informed consent was obtained. The exclusion criteria applied were (1)–(4) and (6), like patients in the PFO migraineurs. Simultaneously, volunteers with a right‐to‐left shunt (screened from healthy volunteers) were included in PFO non‐migraineurs group.

Basic demographic and clinical characteristics were collected from all participants, and dCA assessment, biomarker measurement, and autonomic function evaluation were performed on all participants.

### 2.2 Contrast‐enhanced transcranial Doppler protocol

We performed contrast‐enhanced transcranial Doppler before PFO closure, as previously described.^7^ The insonation of one MCA was accomplished via transcranial Doppler (MultiDop X4, DWL) with the patient in a supine position. An 18‐gauge needle was inserted into the patient's cubital vein, and a mixed contrast agent (9 mL normal saline, 1 mL air, and a drop of the patient's blood) was injected as a rapid bolus. Testing was performed once at rest and twice during the Valsalva maneuver, and the maximum right‐to‐left shunt was recorded. The presence of >10 microbubbles was indicative of a large right‐to‐left shunt or large residual shunting.^12^

### 2.3 Transcatheter PFO closure

Patients in the PFO migraineurs group underwent PFO closure surgery. Procedures were performed by a chief surgeon and involved a femoral approach with the patient under local anesthesia. A long sheath was advanced into the left atrium and an Amplatzer PFO Occluder (AGA Medical Corporation), which was a self‐expanding double‐disk device, was used to occlude the PFO under fluoroscopic guidance, and closure was confirmed via intra‐cardiac echocardiography. Patients were administered heparin (80–100 U/kg) during the procedure and they subsequently underwent routine electrocardiography, contrast transthoracic echocardiography, and chest radiography 24 h after the PFO closure procedure. Aspirin (100 mg/day) alone or in combination with clopidogrel (75 mg/day) was administered as antiplatelet therapy for 6 months following the procedure.

### 2.4 Evaluation of dCA

dCA data were collected from all participants, and PFO migraineurs received two additional dCA measurements, one within 48 h post‐surgery and the other at 1‐month post‐surgery.

dCA was evaluated in a dedicated soundproof room, as previously reported.^14^ With the participant in the supine position, continuous cerebral blood flow velocity (CBFV) and ABP were recorded simultaneously for approximately 10 min in the bilateral MCA using transcranial Doppler (MultiDop X4, DWL), ABP was measured at the fingertip of the middle finger using a servo‐controlled plethysmograph (Finometer Model 1, FMS), respectively. The end‐tidal CO_2_ was also measured using a capnograph (MultiDop X4, DWL) with a face mask attached to the nasal cannula to confirm the stability of respiration during the experiment, in accordance with the recommendations of the dCA monitoring white paper.^15^

The dynamic relationship between the continuous CBFV and ABP was assessed via transfer function analysis (TFA) using MATLAB (Math Works). Evaluation parameters included phase difference (the main parameter, determined as the phase shift angle ranging from 0° to 90°), gain (difference in the amplitude between CBFV and ABP), and the coherence function (indicates signal‐to‐noise ratio). Each parameter was averaged in 0.06–0.12 Hz,^14^ and a cut‐off value for coherence was set at 0.34 to establish the validity of TFA estimates as previously recommended (number of windows: 5; critical values of coherence estimate: α = 5% significant level).^15^ Generally, a lower phase difference indicates an impaired dCA and a phase difference at 0° indicates a CBFV that passively follows ABP changes with no dCA at all. High gain at the same frequency band is also suggestive of compromised dCA for passively transferring the amplitude of ABP to CBFV, though less sensitive than phase difference.^16^

### 2.5 Beat‐to‐beat HRV measurement and analysis

The autonomic function was assessed through beat‐to‐beat HRV analysis. The baseline beat‐to‐beat HRV of each participant was determined while dCA values were measured. Then, PFO migraineurs underwent two additional HRV measurements: one within the 48‐h period following surgery and the other 1 month after surgery. Continuous recordings of beat‐to‐beat information obtained from the servo‐controlled plethysmograph were processed using MATLAB scripts developed by our research team, as previously reported.^17^ Ectopic beats and artifacts were automatically detected, visually confirmed, and removed via linear interpolation. The software processed the beat‐to‐beat recordings and generated heart period values. HRV was calculated using Heart Rate Variability Software in MATLAB designed by Ramshur in 2010 (https://sourceforge.net/projects/hrvas/). The time‐domain analysis of HRV comprised normal‐to‐normal (NN) intervals, standard deviation of all NN intervals (SDNN), and square root of the mean of the sum of squares of differences between adjacent NN intervals (RMSSD). The spectral power of NN intervals was calculated using the Welch method, based on a fast‐Fourier transform. HRV variables were analyzed in the frequency domain, and total power (TP), power of very‐low‐frequency (VLF; <0.04 Hz), low‐frequency (LF; 0.04–0.15 Hz), high‐frequency (HF; 0.15–0.40 Hz), and LF to HF ratios (LF/HF) were assessed.^17^ LF and HF variables were expressed in ms^2^ and normalized units (nu), calculated using the following formula: nLF or nHF = LF or HF/(TP–VLF) × 100. The SDNN, RMSSD, TP, VLF, LF (ms^2^), and HF (ms^2^) components were log‐transformed to ensure normal distribution of parameters. Individuals with data involving ectopic beats occurring at a rate > 20% during HRV measurements were excluded.^18^

### 2.6 Blood biomarker measurement

Human specimens were obtained from the Department of Biobank, Division of Clinical Research, the first hospital of Jilin University. Arterial blood (obtained from radial artery) and venous blood (obtained from the cubital vein/basilic vein) were collected in all participants, and arterial blood was collected again immediately post‐surgery from the PFO migraineurs. Twenty stroke‐related blood biomarkers were analyzed with the Quantibody Human custom array from RayBiotech using a quantitative cytokine chip as previously reported.^19^, ^20^, ^21^, ^22^, ^23^, ^24^, ^25^, ^26^, ^27^, ^28^, ^29^, ^30^, ^31^, ^32^, ^33^, ^34^, ^35^, ^36^ A list of the 20 biomarkers as well as their relationships with stroke is shown in Table 1. The measurement was performed according to the manufacturer's instructions, and four technical replicates were applied for each sample. A GenePix 4000B laser scanner (Bio‐Rad Laboratories) was used to capture signals (green fluorescence, Cy3 channel, 555 nm excitation, and 565 nm emission). We analyzed the images using GenePix Pro 6.0 microarray analysis software and quantified the levels of biomarkers according to the standard curve calibrated from the same array.

**TABLE 1:** Twenty arterial blood biomarkers and their relationship with stroke.

Biomarkers	Relationship with stroke
Angiogenin	An indicator of endothelial damage, related to the progression of vascular disease^19^
Angiotensinogen, Renin	Components of the renin‐angiotensin system, implicated in atherosclerosis and associated with increased risk for ischemic stroke^20^
CRP	An acute phase reactant appears to be predictive of stroke risk^21^
lgG	Provides important information on the humoral immune status and has been reported to be associated with the occurrence of cardio‐cerebrovascular disease events^22^
IL‐1β, IL‐6	Lymphatic factors associated with the risk of ischemic stroke^23^, ^24^
Kallikrein 5	A component of the kallikrein‐kinin system that protects against vascular injury and stroke^25^
LOX‐1	A transmembrane endocytosis receptor of oxidized low‐density lipoprotein involved in the development of atherosclerotic disease, which is increased in ischemic stroke and transient ischemic attack^26^
MIP‐1α	Plays a potentially important role in the development of inflammatory responses and plays a significant role in the etiopathogenesis of cardiovascular disease^27^
MMP‐2, MMP‐9	The two best‐studied matrix metalloproteinases, increase of which results in aberrant proteolysis contributing to blood–brain barrier dysfunction and in part determining the extent of the infarct^28^
PAI‐I, t‐PA	Components of the fibrinolysis system; PAI‐I inhibits the activity of t‐PA, and t‐PA activates plasminogen; imbalance of the system leads to stroke^29^
PDGF‐BB	One of the most potent stimulants promoting the development of atherosclerosis^30^
S100b	A reliable biomarker of neural injury including stroke^31^
Tau	Involved in the regulation of blood–brain barrier integrity, and opening of the blood–brain barrier is implicated in stroke^32^
TNF‐α	A proinflammatory and proatherogenic cytokine that modulates tissue injury in stroke^24^, ^33^
VEGF‐a	Participates in atherosclerosis, neuroprotection, neurogenesis, and angiogenesis; its decrease leads to endothelial dysfunction and increased risk of cardio‐cerebrovascular disorders^19^, ^34^
VE‐Cadherin	An endothelial‐specific adhesion protein at adherens junctions, related to vascular integrity and associated with progressive ischemic stroke^35^, ^36^

Abbreviations: CRP, C reactive protein; IL, interlukin; lgG, immunoglobulin; LOX‐1, lectin‐like oxidized low‐density lipoprotein receptor‐1; MIP‐1α, macrophage inflammatory protein‐1α; MMP‐2, matrix metalloproteinase‐2 MMP‐9; PAI‐I, plasminogen activator inhibitor‐I; PDGF‐BB, platelet‐derived growth factor‐BB; p‐TA, tissue‐type plasminogen activator; TNF‐α, tumor necrosis factor‐α; VE‐Cadherin, vascular endothelial‐Cadherin; VEGF‐a, vascular endothelial growth factor‐a.

### 2.7 Statistical analysis

Data were analyzed using Statistical Program for Social Sciences version 19.0 (SPSS, IBM Corp.). The distribution of data was assessed using a one‐sample Kolmogorov–Smirnov test and numerical variables were analyzed based on normality. Normally distributed data are expressed as mean ± standard deviation (SD) and analyzed with one‐way ANOVA or Student's *t*‐test. Non‐normally distributed data are expressed as median (interquartile range) and analyzed with Wilcoxon signed‐rank test (for paired continuous data) or Mann–Whitney test (for independent data). A general linear model was used to detect differences among PFO migraineurs of repeated measurements (before, after, and 1‐month post‐closure), as deemed appropriate based on data distributions. Comparisons were corrected post hoc via Bonferroni adjustment. Enumeration variables are expressed as absolute values and percentages and compared using the chi‐square test or Fisher's exact test. Calculated two‐tailed *p*‐values < 0.05 were considered significant.

## 3 RESULTS

### 3.1 Demographic information

**FIGURE 1:**
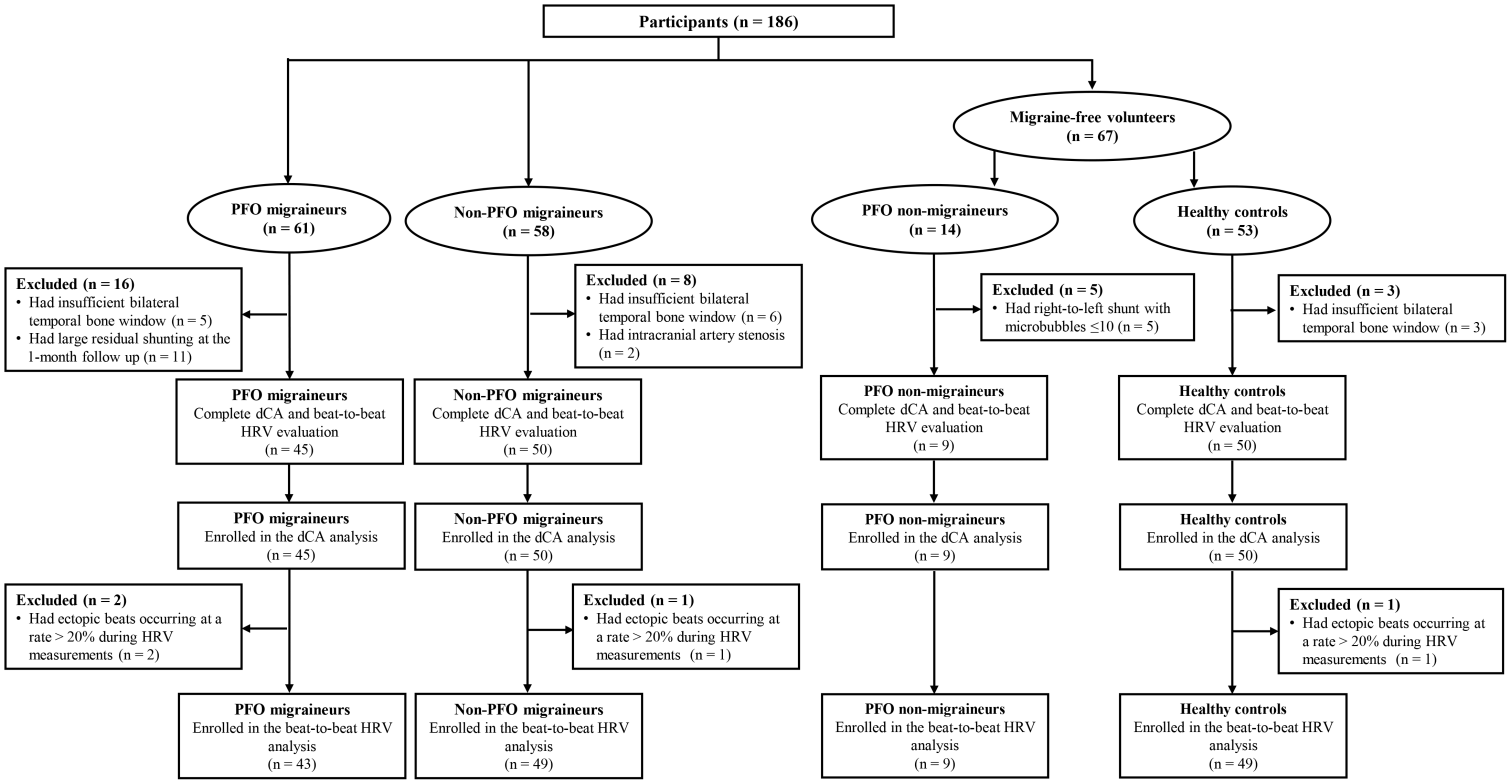
The flowchart of the study. dCA, dynamic cerebral autoregulation; HRV, heart rate variability; PFO, patent foramen ovale.

Of the 61 PFO migraineurs, five had an insufficient bilateral temporal bone window and 11 had a large residual shunting at the 1‐month follow‐up; these patients were excluded. Thus, a total of 45 PFO migraineurs were included. Of the 58 non‐PFO migraineurs, six had an insufficient bilateral temporal bone window, and two had intracranial artery stenosis. Finally, a total of 50 non‐PFO migraineurs were included. In addition, after screening 67 age‐ and sex‐matched migraine‐free volunteers, 14 had a right‐to‐left shunt and three had an insufficient bilateral temporal bone window. Of these, 14 PFO volunteers, nine had a large right‐to‐left shunt. Thus, 50 healthy controls and 9 PFO non‐migraineurs were included. The flowchart was shown in Figure 1. No participants lost follow‐up. Since the sample size of PFO without the migraine group is really small, we put the related statistical analyses and results of this group in the Supplementary Materials (Tables S1–S3).

The demographic and clinical information of PFO migraineurs, non‐PFO migraineurs, and healthy controls are presented in Table 2. No significant differences were found in age, sex, comorbidities such as hypertension, hyperlipidemia, diabetes, current smoking status, mean arterial pressure, or end‐tidal CO_2_ between the groups.

**TABLE 2:** Demographic and clinical information of participants.

	PFO migraineurs (*n* = 45)	Non‐PFO migraineurs (*n* = 50)	Healthy controls (*n* = 50)	*F*	*p*
Age, years old	37.69 ± 12.26	37.36 ± 12.28	36.64 ± 10.63	0.101	0.904
*Age groups, n (%)*				0.137	0.998
≥18, <35	20 (44.44)	21 (42.00)	22 (44.00)		
≥35, <50	18 (40.00)	20 (40.00)	20 (40.00)		
≥50, <65	7 (15.56)	9 (18.00)	8 (16.00)		
Sex, male, *n* (%)	13 (28.89)	16 (32.00)	15 (30.00)	0.113	0.945
Hypertension, *n* (%)	7 (15.60)	6 (12.00)	7 (14.00)	0.255	0.880
Hyperlipidemia, *n* (%)	9 (20.00)	9 (18.00)	11 (22.00)	0.250	0.882
Diabetes mellitus, *n* (%)	5 (11.11)	4 (8.00)	4 (8.00)	0.368	0.832
Current smoking, *n* (%)	10 (22.22)	10 (20.00)	13 (26.00)	0.523	0.770
Mean arterial pressure, mmHg	88.78 ± 7.24	88.10 ± 5.32	87.10 ± 8.23	0.689	0.504
End‐tidal CO_2_, mmHg	40.82 ± 4.64	40.34 ± 3.17	39.50 ± 5.38	1.064	0.348

Abbreviation: PFO: patent foramen ovale closure.

Two PFO migraineurs, one non‐PFO migraineur, and one healthy control presented with ectopic beats occurring at a frequency > 20% during HRV measurement, and were therefore excluded from the HRV analysis. Forty‐five paired arterial blood samples (before and after PFO closure), 42 venous blood samples from PFO migraineurs, and both arterial and venous blood samples from 16 healthy controls (the rest of the volunteers were reluctant to undergo venipuncture) were assessed for 20 stroke‐related arterial blood biomarkers.

### 3.2 Dynamic cerebral autoregulation evaluation

**FIGURE 2:**
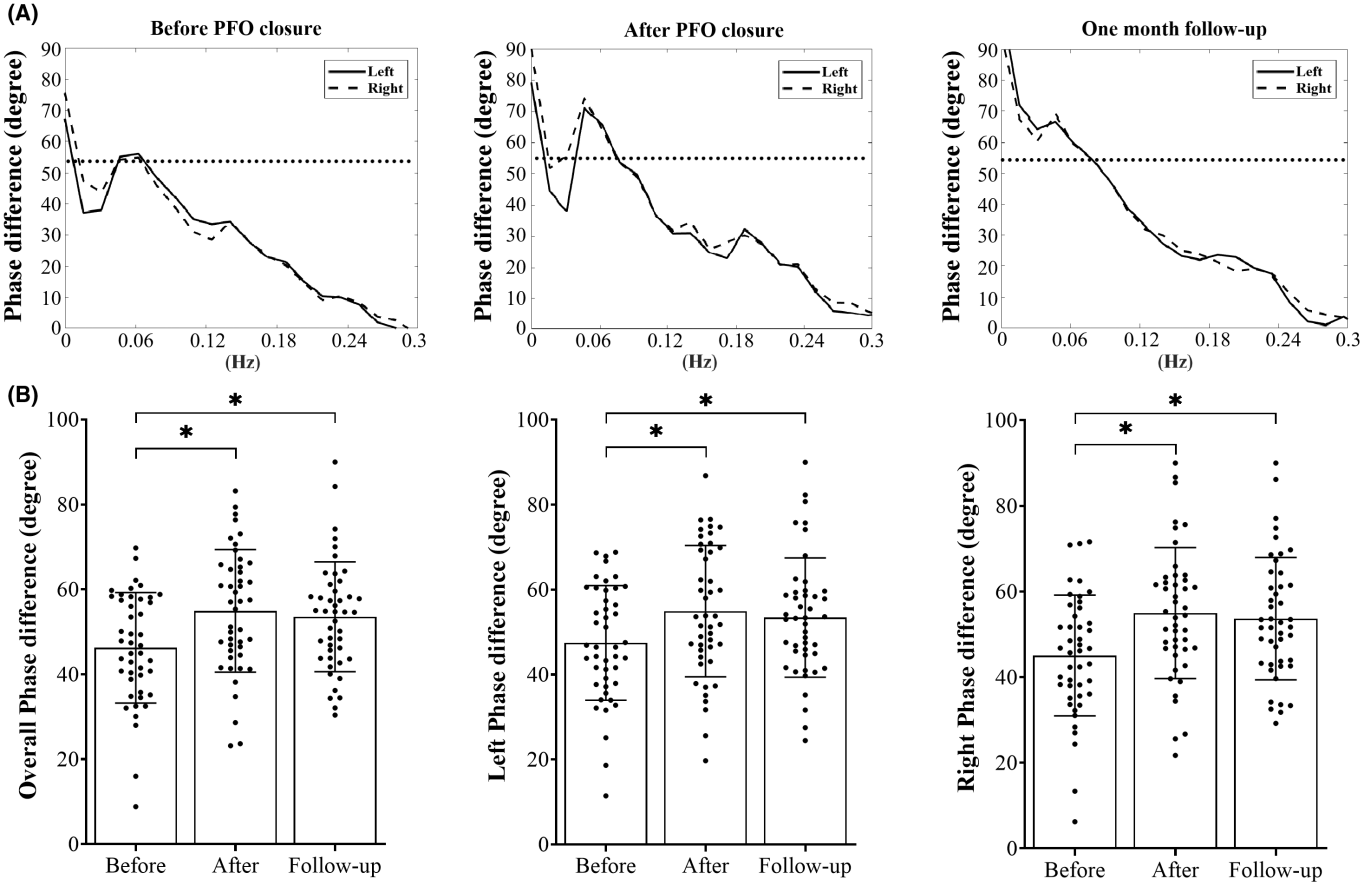
The autoregulatory parameter (phase difference) determined in patent foramen ovale (PFO) patients pre‐ and post‐closure. (A) The phase difference derived from the transfer function analysis. Dashed horizontal line represents the mean value of phase difference in the control group. (B) Phase differences in PFO migraineurs before and after surgery (mean ± standard deviation). *p < 0.05 between two groups.

As shown in Table 3, the PFO migraineurs exhibited significantly lower phase differences in the left hemisphere, the right hemisphere, and the whole brain overall (combined left and right hemispheres) compared to non‐PFO migraineurs and healthy controls. Phase differences in PFO migraineurs post‐surgery and at 1‐month follow‐up showed no significant difference when compared with those of healthy controls, indicating that dCA were largely resolved after PFO closure, and eventually reached the levels of dCA observed in the control group. These results remained unchanged at 1‐month post‐surgery (Figure 2). No significant difference was found in the gain between the three groups, neither before PFO closure, nor PFO closure nor 1‐month post‐surgery (Table 3).

**TABLE 3:** Comparison of phase differences and gains in different groups.

	PFO migraineurs (*n* = 45)	Non‐PFO migraineurs (*n* = 50)	Healthy controls (*n* = 50)	*F*	*p*
*Phase difference (degree)*					
Overall	46.25 ± 13.02^a^, ^b^	54.19 ± 8.95	54.46 ± 14.28	6.727	0.002
Left hemisphere	47.45 ± 13.49^a^, ^b^	54.36 ± 9.85	54.26 ± 16.03	4.084	0.019
Right hemisphere	45.05 ± 14.10^a^, ^b^	54.02 ± 9.50	54.67 ± 14.54	8.117	<0.001
*Gain (%/mmHg)*					
Overall	1.15 ± 0.36	1.09 ± 0.30	1.23 ± 0.24	2.847	0.061
Left hemisphere	1.13 ± 0.34	1.07 ± 0.29	1.21 ± 0.26	2.762	0.067
Right hemisphere	1.16 ± 0.40	1.10 ± 0.31	1.25 ± 0.27	2.463	0.089

	PFO migraineurs			*F*	*p*
	Before closure (*n* = 45)	After closure (*n* = 45)	One month follow‐up (*n* = 45)		
*Phase difference (degree)*					
Overall	46.25 ± 13.02^c^	54.94 ± 14.44^d^	53.78 ± 13.68^d^	8.786	<0.001
Left hemisphere	47.45 ± 13.49^c^	54.93 ± 15.47^d^	53.67 ± 14.74^d^	5.840	0.006
Right hemisphere	45.05 ± 14.10^c^	54.95 ± 15.30^d^	53.89 ± 14.96^d^	8.570	<0.001
*Gain (%/mmHg)*					
Overall	1.15 ± 0.36	1.04 ± 0.28^c^	1.14 ± 0.35	1.516	0.231
Left hemisphere	1.13 ± 0.34	1.04 ± 0.30^c^	1.12 ± 0.36	1.080	0.349
Right hemisphere	1.16 ± 0.40	1.05 ± 0.28^c^	1.16 ± 0.35	1.924	0.158

Abbreviation: PFO, patent foramen ovale.

^a^ *p* < 0.05 for comparison with non‐PFO migraineurs, with post hoc analysis performed by Bonferroni's method.

^b^ *p* < 0.05 for comparison with healthy controls, with post hoc analysis performed by Bonferroni's method.

^c^ *p* < 0.05 for comparison with healthy controls using Student's *t*‐test.

^d^ *p* < 0.05 for comparison with before closure, with post hoc analysis performed by Bonferroni's method.

### 3.3 Blood biomarkers

#### 3.3.1 Platelet‐derived growth factor‐BB

The arterial PDGF‐BB levels were higher in PFO migraineurs than in healthy controls, which were immediately corrected by PFO closure (Table 4). And venous PDGF‐BB levels were lower in PFO migraineurs than in healthy controls (646.33 [489.76–896.69] vs. 958.50 [821.12–1281.46], *p* = 0.002).

**TABLE 4:** Biomarkers in the arterial blood in migraineurs with patent foramen ovale (before and after closure) and controls.

Biomarkers	PFO migraineurs		Healthy controls (*n* = 16)
	Before closure (*n* = 45)	After closure (*n* = 45)	
Angiogenin	247.9 (177.6, 359.1)	237.3 (199.0, 308.5)	302.8 (245.4, 334.1)
Angiotensinogen	15200.1 (9528.6, 21980.3)	14401.8 (8662.0, 21726.2)	17738.1 (10912.5, 28879.4)
CRP	13443.3 (10506.0, 19728.8)^a^	13649.0 (9815.9, 19465.5)^a^	20267.2 (15425.7, 27860.3)
lgG	53840.3 (28283.9, 93866.1)	47886.5 (25686.1, 74937.5)	61128.4 (48452.3, 108586.7)
IL‐1β	226.4 (128.4, 347.8)	206.8 (112.4, 322.7)	289.8 (190.9, 363.8)
IL‐6	392.9 (309.6, 524.1)	393.3 (284.9, 509.1)	428.7 (328.0, 588.0)
Kallikrein 5	3280.8 (2265.1, 4677.2)^a^	3152.5 (1707.9, 5204.6)^a^	4703.4 (2981.1, 7742.3)
LOX‐1	405.7 (253.2, 850.6)	473.3 (260.4, 956.8)	557.6 (443.1, 796.8)
MIP‐1α	686.8 (493.0, 1108.3)	670.3 (496.2, 939.8)	558.7 (406.7, 667.5)
MMP‐2	166404.8 (123678.6, 257657.2)^a^	171067.8 (120173.0, 248469.1)^a^	233275.7 (189532.5, 267317.9)
MMP‐9	6396.7 (4372.9, 8554.1)	5997.1 (4271.6, 7285.0)	5251.0 (3497.2, 6174.9)
PAI‐I	20469.8 (13971.5, 25204.8)	18880.4 (15355.3, 24764.7)	20585.9 (15055.9, 29360.4)
PDGF‐BB	624.4 (453.2, 827.7)^a^	531.2 (302.8, 736.5)^a^, ^b^	111.0 (81.7, 151.4)
Renin	7444.6 (4156.8, 11737.9)	6884.0 (4446.1, 9850.0)	6062.7 (5135.2, 7533.5)
S100b	122372.4 (78750.4, 199832.9)^a^	121333.5 (82535.1, 189602.4)^a^	196010.5 (138298.5, 255105.5)
Tau	116899.8 (86631.7, 142477.6)^a^	114275.1 (85492.4, 149115.7)^a^	152607.6 (112969.7, 194483.5)
TNF‐α	8625.8 (6295.0, 11935.5)	8152.7 (6745.2, 12763.7)	12703.2 (8031.7, 16651.5)
t‐PA	5891.5 (3807.0, 9724.0)^a^	6762.5 (4247.1, 10978.4)	9830.6 (6050.9, 11921.0)
VEGF‐a	997.5 (718.3, 2006.7)^a^	1086.0 (709.1, 2080.8)^a^	1742.2 (1355.0, 3049.3)
VE‐Cadherin	107104.7 (62260.7, 266435.1)	137877.1 (76581.0, 257124.1)	133380.9 (107067.9, 200629.1)

Abbreviations: CRP, C‐reactive protein; IgG, immunoglobulin G; IL‐1β, interleukin‐1β; IL‐6, interleukin‐6; LOX‐1, lectin‐like oxidized low‐density lipoprotein receptor‐1; MIP‐1α, macrophage inflammatory protein‐1α; MMP‐2, matrix metalloproteinase‐2; MMP‐9, matrix metalloproteinase‐9; PAI‐I, plasminogen activator inhibitor‐I; PDGF‐BB, platelet‐derived growth factor‐BB; PFO, patent foramen ovale; TNF‐α, tumor necrosis factor‐α; t‐PA, tissue‐type plasminogen activator; VE‐cadherin, vascular endothelial‐cadherin; VEGF‐a, vascular endothelial growth factor‐a.

^a^ *p* < 0.05 compared with healthy controls using Mann–Whitney test.

^b^ *p* < 0.05 compared with before closure using Wilcoxon signed‐rank test.

**FIGURE 3:**
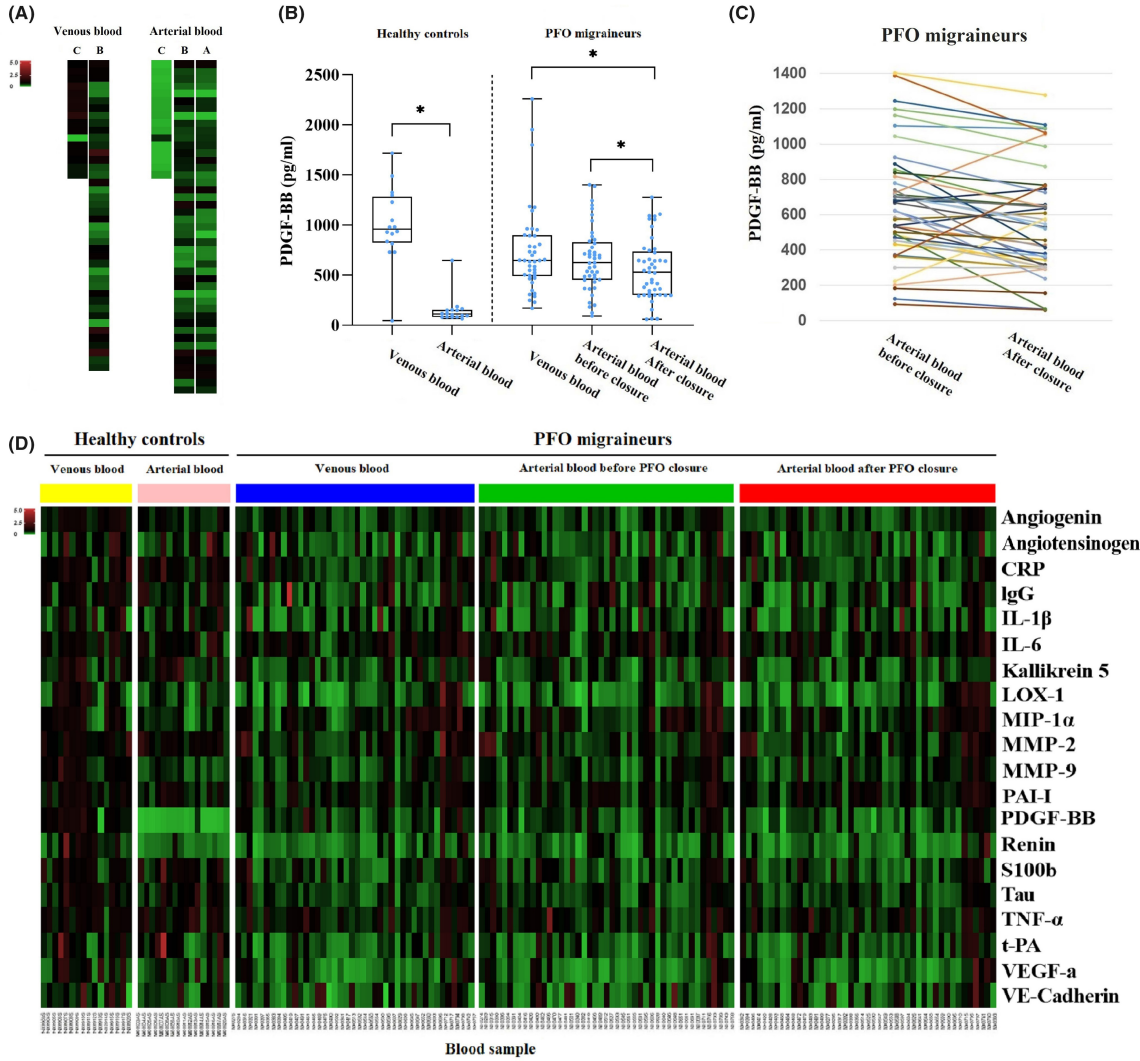
Expression of 20 biomarkers in the venous and arterial blood. (A) Heatmap of venous and arterial relative PDGF‐BB levels in healthy controls and PFO migraineurs (relative to mean venous PDGF‐BB levels of the healthy control). Panel (C) represented healthy controls, Panel (B) represented before closure, A represented after closure. (B) Box plot with individual dot of venous and arterial absolute PDGF‐BB levels in healthy controls and PFO migraineurs. (C) Changes of arterial absolute PDGF‐BB levels in each subject after PFO closure. (D) Heatmap of relative levels of 20 biomarkers in the venous and arterial blood (relative to mean venous level of each biomarker of the healthy control). *p < 0.05 for comparison with two groups. CRP, C‐reactive protein; IgG, immunoglobulin G; IL‐1β, interleukin‐1β; IL‐6, interleukin‐6; LOX‐1, lectin‐like oxidized low‐density lipoprotein receptor‐1; MIP‐1α, macrophage inflammatory protein‐1α; MMP‐2, matrix metalloproteinase‐2; MMP‐9, matrix metalloproteinase‐9; PAI‐I, plasminogen activator inhibitor‐I; PDGF‐BB, platelet‐derived growth factor‐BB; PFO, patent foramen ovale; TNF‐α, tumor necrosis factor‐α; t‐PA, tissue‐type plasminogen activator; VE‐cadherin, vascular endothelial‐cadherin; VEGF‐a, vascular endothelial growth factor‐a.

Interestingly, we observed that in the control group, the venous PDGF‐BB level was about 8‐fold higher than the arterial level [958.5 (821.1, 1281.5) vs. 111.0 (81.7, 151.4), Figure 3A,B]. Such dramatic difference was absent in PFO migraineurs [venous vs. arterial: 646.3 (489.8, 896.7) vs. 622.8 (446.8, 822.4), Figure 3A,B], possibly due to the abnormally high arterial PDGF‐BB levels. Immediately after PFO closure, the arterial PDGF‐BB levels in PFO migraineurs decreased significantly (Figure 3C and Table 4), likely due to that venous blood entering the artery through the foramen ovale resulting in increased PDGF‐BB levels in the arterial blood, which can be partly alleviated with PFO closure. However, PDGF‐BB did not drop to the level of the normal control group after PFO closure, indicating that there are other factors causing PDGF‐BB level increase in PFO migraineurs.

#### 3.3.2 Other biomarkers

The levels of angiogenin, angiotensinogen, CRP, IgG, IL‐1β, IL‐6, kallikrein 5, LOX‐1, MIP‐1α, MMP‐2, MMP‐9, PAI‐I, renin, S100b, Tau, TNF‐α, t‐PA, VEGF‐a, and VE‐cadherin in arterial blood did not change significantly after PFO closure (Figure 3D and Table 4).

### 3.4 Beat‐to‐beat heart rate variability

No significant differences in HRV values were observed between the healthy controls and PFO migraineurs (Table 5). However, in the PFO migraineurs, time‐domain HRV indices, NN intervals, SDNN, and RMSSD values were significantly lower after PFO closure compared to pre‐closure baseline. Absolute frequency domain parameters, including TP, VLF, LF, and HF, were synchronously reduced after PFO closure. These changes indicated that PFO closure transiently altered the cardiac autonomic function. Fortunately, these parameters had been fully restored to the preoperative levels by 1‐month post‐closure. Notably, the nLF, nHF, and LF/HF values did not change during the process, indicating a stable balance between the sympathetic and parasympathetic activities (Table 5).

**TABLE 5:** Comparison of beat‐to‐beat heart rate variability parameters in different groups.

	PFO migraineurs (*n* = 43)	Non‐PFO migraineurs (*n* = 49)	Healthy controls (*n* = 49)	*F*	*p*
*Time domain indices*					
NN intervals (ms)	864.89 ± 106.38	832.28 ± 140.07	834.32 ± 106.46	1.053	0.352
SDNN (ms, log)	1.58 ± 0.18	1.55 ± 0.18	1.61 ± 0.15	1.326	0.269
RMSSD (ms, log)	1.52 ± 0.23	1.48 ± 0.22	1.54 ± 0.18	1.007	0.368
*Frequency domain indices*					
TP (ms^2^, log)	3.06 ± 0.39	3.02 ± 0.39	3.14 ± 0.30	1.331	0.267
VLF (ms^2^, log)	2.66 ± 0.40	2.59 ± 0.44	2.66 ± 0.31	0.475	0.623
LF (ms^2^, log)	2.34 ± 0.44	2.40 ± 0.39	2.50 ± 0.36	1.788	0.171
HF (ms^2^, log)	2.55 ± 0.49	2.48 ± 0.49	2.68 ± 0.40	2.442	0.091
LF (nu.)	39.71 ± 15.58	45.45 ± 16.18	40.66 ± 15.70	1.786	0.171
HF (nu.)	60.29 ± 15.58	54.55 ± 16.18	59.34 ± 15.70	1.786	0.171
LF/HF	0.93 ± 0.12	0.99 ± 0.19	0.94 ± 0.11	2.318	0.102

	PFO migraineurs			*F*	*p*
	Before closure (*n* = 43)	After closure (*n* = 43)	One month follow‐up (*n* = 43)		
*Time domain indices*					
NN intervals (ms)	864.89 ± 106.38	782.40 ± 123.58^a^, ^b^	846.05 ± 99.12	13.16	<0.001
SDNN (ms, log)	1.58 ± 0.18	1.43 ± 0.19^a^, ^b^	1.52 ± 0.17^b^	14.41	<0.001
RMSSD (ms, log)	1.52 ± 0.23	1.40 ± 0.22^a^, ^b^	1.46 ± 0.22	5.19	0.008
*Frequency domain indices*					
TP (ms^2^, log)	3.06 ± 0.39	2.69 ± 0.42^a^, ^b^	2.94 ± 0.37^b^	20.51	<0.001
VLF (ms^2^, log)	2.66 ± 0.40	2.36 ± 0.39^a^, ^b^	2.56 ± 0.35	10.59	<0.001
LF (ms^2^, log)	2.34 ± 0.44	1.93 ± 0.53^a^, ^b^	2.24 ± 0.44^b^	19.41	<0.001
HF (ms^2^, log)	2.55 ± 0.49	2.09 ± 0.54^a^, ^b^	2.39 ± 0.52^b^	18.90	<0.001
LF (nu.)	39.71 ± 15.58	42.04 ± 21.45	42.47 ± 18.07	0.41	0.67
HF (nu.)	60.29 ± 15.58	57.96 ± 21.45	57.53 ± 18.07	0.41	0.67
LF/HF	0.93 ± 0.12	0.94 ± 0.23	0.95 ± 0.16	0.31	0.73

Abbreviations: HF, high‐frequency; HRV, heart rate variability; LF, low‐frequency; NN intervals: time interval between successive heartbeats from which artifacts have been removed; PFO, patent foramen ovale; RMSSD: the root mean square of successive differences of NN intervals; SDNN: the standard deviation of all NN intervals; TP, total power; VLF, very‐low frequency.

^a^ *p* < 0.05 for comparison with before closure, with post hoc analysis performed by Bonferroni's method.

^b^ *p* < 0.05 for comparison with healthy controls using Student's *t*‐test.

## 4 DISCUSSION

In this study, we report that dCA was impaired in migraine patients with PFO, which was promptly and persistently improved upon PFO closure. PFO closure could also immediately and significantly restore the abnormally high levels of PDGF‐BB in the arterial blood. The above findings may be related to the pathogenesis of stroke occurrence in migraine patients, and PFO closure may restore these changes to prevent stroke occurrence and recurrence. In addition, PFO closure could cause temporary abnormalities in cardiac autonomic function that are entirely restored 1 month later.

Previous studies reported that the risk of stroke was increased in individuals with migraine [relative risk 2.16; 95% confidence interval (CI) 1.89–2.48].^37^ The pathogenesis of this phenomenon is not clear. PFO has been a hot topic in recent years, and its relationship with migraine and stroke deserves attention. In the present study, we focused on migraine patients with PFO, a special type of migraine, tried to explain the relationship between PFO and stroke from the perspective of the pathophysiological changes caused by PFO, and observed the effect of PFO closure on these pathophysiological changes. We did this study on migraine patients for two main reasons. First, the incidence of PFO in migraine patients is approximately 14.6%–66.5%, while the incidence in the general population is 9%–27.3%.^38^ In this way, it is more efficient to screen out patients with PFO among migraine patients. Second, if patients with PFO do not have any symptoms, there may be ethical issues with PFO closure.

Owing to dCA, constant cerebral blood flow could be maintained despite changes in cerebral perfusion pressure and ABP. dCA is an important indicator of a normal cerebrovascular functioning.^14^ As Caplan reported, poor cerebrovascular function could diminish the ability of the cerebral circulation to clear thromboemboli and limit the availability of blood flow to the ischemic regions,^6^ and thus a dysfunction in dCA may increase an individual's susceptibility to stroke. In the present study, we confirmed the presence of dCA impairment in patients with PFO. Moreover, as shown in Table S2, although the sample size was small, dCA of PFO patients without migraine was significantly lower than those measured in migraine patients without PFO and healthy control. Therefore, we speculated that migraine may be not associated with dCA alteration. And our results were in line with the previous study.^39^, ^40^ The possible mechanism of impaired dCA in PFO patients may involve cortical spreading depression induced by cerebral arterial microemboli originating from the venous system, which further results in impaired dCA by affecting vasomotor function.^41^ Furthermore, we found that PFO closure could reverse the impaired dCA in patients with PFO, thus supporting the notion that PFO migraineurs could benefit from PFO closure.

In the analysis of blood biomarkers, we found dramatically high arterial PDGF‐BB levels, and an altered arteriovenous PDGF‐BB ratio in PFO migraineurs. Several in vivo and in vitro studies have shown that PDGF‐BB is one of the most potent stimulants for vascular smooth muscle cell proliferation. PDGF‐BB is also potent in transforming vascular smooth muscle cells from a contractile phenotype to a synthetic phenotype, a critical step in the development of atherosclerosis.^30^ The TOSS‐2 trial (Trial of Cilostazol in Symptomatic Intracranial Stenosis‐2) reported that the venous PDGF‐BB level is associated with the progression of symptomatic intracranial atherosclerotic stenosis.^42^ And a study found that baseline venous PDGF‐BB was higher in stroke patients than in controls.^43^ Furthermore, PDGF‐BB acts as a vasoconstrictor^44^ causing severe and long‐lasting vasoconstriction, a direct cause of dCA impairment leading to unstable cerebral blood flow. We speculated that all these factors working together contribute to susceptibility to stroke.^30^, ^42^, ^43^ Venous blood was used in all the above studies and the characteristics of PDGF‐BB in arterial blood are not been reported. In our study, we compared the levels of PDGF‐BB in the arterial blood of patients with PFO before and after surgery for the following considerations: PDGF‐BB in venous blood may be shunt to arterial blood in patients with PFO, resulting in abnormal changes of arterial PDGF‐BB. Given PDGF‐BB particularly acts on vascular smooth muscle cells, while arteries contain more vascular smooth muscle cells, arterial blood PDGF‐BB may better reflect the true state of PDGF‐BB; in addition, after PFO closure, following the disappearance of venoarterial shunt, PDGF‐BB levels in arterial blood may be partially recovered. Therefore, the changing trend of PDGF‐BB before and after PFO closure can be visually detected in arterial blood. In the present study, we did observe significant changes in arterial blood PDGF‐BB in PFO patients before and after PFO closure, and we considered these patients may benefit from PFO closure. Additionally, we compared venous PDGF‐BB levels between controls and PFO patients and found venous PDGF‐BB levels were lower in PFO patients than in controls. The causes for this phenomenon are unclear, one possibility is that as the venous blood is shunted into arterial blood, the PDGF‐BB content is also partially transferred from the vein to the artery, resulting in lower PDGF‐BB levels in venous blood.

Furthermore, as known, the lung is located between the venous blood and the arterial blood, and a dramatic drop in PDGF‐BB is observed in the arterial blood in healthy controls. It is reasonable to suspect that the lung could somewhat eliminate the PDGF‐BB through unknown mechanisms. PFO patients with large right‐to‐left shunt typically have pulmonary congestion, pulmonary hypertension, and/or dysfunctional pulmonary microcirculation,^45^ which also affect the elimination of PDGF‐BB from the lung, which may explain the fact that postoperative PDGF‐BB does not fall to the level of the normal control group.

By analyzing cardiac autonomic function, we found that there were no significant changes in HRV values of migraine patients with PFO, indicating that autonomic dysfunction may not be involved in the corresponding stroke susceptibility. Notably, we observed that time‐domain and absolute frequency‐domain HRV after PFO closure decreased significantly, consistent with the previous findings.^46^ The immediate post‐surgical HRV alteration and autonomic dysfunction are likely caused by cardiac surgery.^47^ Notably, the HRV‐related parameters recovered to the preoperative levels after 1 month, indicating that the effect of PFO closure on the autonomic nervous system was essentially transient. Interestingly, the nLF, nHF, and LF/HF values did not change during the process, indicating that PFO closure did not alter the balance between the sympathetic and parasympathetic activities.

This study had some limitations. First, because of patient intolerance, we did not collect venous and arterial blood samples throughout the long‐term follow‐up from patients who underwent PFO closure. Second, the sample size in our study was relatively small. Thus, our findings warrant further investigation using large‐scale studies. Third, changes in beat‐to‐beat variability may not directly and comprehensively reflect cardiac autonomic changes, therefore, the findings of cardiac autonomic regulation in our study should be further verified using other methods such as plasma markers.^48^

In conclusion, PFO closure can improve dCA and alter elevated arterial blood PDGF‐BB levels in migraine patients with PFO, both of which may be related to the preventive effect of PFO closure on stroke occurrence and recurrence in these patients.

## AUTHOR CONTRIBUTIONS

YY and Z‐NG drafted the initial protocol, which was reviewed with critical revisions and approval by all authors. YQ and PZ did the statistical analysis. YY, Z‐NG, JL, and JC wrote the first draft of the manuscript. YG, YX, HM, Y‐ZG, HJ, XS, and QH collected data. KH, H‐HH, and DMS revised the manuscript. All authors contributed to data acquisition. All authors contributed to the critical revision of the manuscript and approved the final manuscript for submission.

## CONFLICT OF INTEREST STATEMENT

The authors declare no conﬂict of interest.

## FUNDING INFORMATION

This work was supported by the National Natural Science Foundation of China (Grant No. 82071291), the Norman Bethune Health Science Center of Jilin University (2022JBGS03), the Science and Technology Department of Jilin Province (YDZJ202302CXJD061), and the Jilin Provincial Key Laboratory (YDZJ202302CXJD017) to YY, and the Norman Bethune Program of Jilin University (2022B02) to Z‐NG.

## PATIENT CONSENT STATEMENT

Written informed consent was obtained from all participants.

## Supporting information


Tables S1‐S3.

## Data Availability

The data that support the findings of this study are available from the corresponding author upon reasonable request.
