# Supplementary material for: Changes in cerebral autoregulation, stroke‐related blood biomarkers, and autonomic regulation after patent foramen ovale closure in severe migraine patients
Source: CNS Neurosci Ther. 2023 May 8;29(10):3031–42. doi: 10.1111/cns.14244 (PMC10493653; doi:10.1111/cns.14244)
Supplement: Supplementary file 1 — Tables S1‐S3. [file CNS-29-3031-s001.docx]

As we reported in the present study, after screening 67 age- and sex-matched migraine-free volunteers, 14 had right-to-left shunt and 3 had an insufficient bilateral temporal bone window. Of these 14 PFO volunteers, 9 had a large right-to-left shunt. Thus, 50 healthy controls and 9 PFO non-migraineurs were ultimately included. Since the sample size of PFO without migraine group is small, we put the statistical analysis and results of this group in the supplementary materials (Table S1, S2, and S3). The baseline characteristics of the four groups (PFO migraineurs, non-PFO migraineurs, PFO non-migraineurs, and healthy controls) were shown in Table S1 and no significant differences was found among the groups. As shown in Table S2, the difference of phase differences in the left hemisphere, right hemisphere, and the whole brain overall (combined left and right hemispheres) was significance. Phase differences of PFO non-migraineurs was significantly lower than those measured in non-PFO migraineurs and healthy control. And when compared to PFO migraineurs, PFO non-migraineurs had similar phase difference levels. As for gain values among four groups, no difference was found. In terms of beat-to-beat HRV parameters, there was no significant differences among the groups (Table S3).

**Table S1. Demographic and clinical information of participants.**

|  | **PFO migraineurs**  **(n=45)** | **Non-PFO migraineurs (n=50)** | **PFO non-migraineurs (n=9)** | **Healthy controls (n=50)** | **F** | ***P*** |
| --- | --- | --- | --- | --- | --- | --- |
| **Age, years old** | 37.69±12.26 | 37.36±12.28 | 38.11±9.27 | 36.64±10.63 | 0.085 | 0.968 |
| **Age groups, n (%)** |  |  |  |  | 0.337 | 0.999 |
| ≥18, <35 | 20 (44.44) | 21 (42.00) | 4 (44.44) | 22 (44.00) |  |  |
| ≥35, <50 | 18 (40.00) | 20 (40.00) | 4 (44.44) | 20 (40.00) |  |  |
| ≥50, <65 | 7 (15.56) | 9 (18.00) | 1 (11.11) | 8 (16.00) |  |  |
| **Sex, male, n (%)** | 13 (28.89) | 16 (32.00) | 5 (55.56) | 15 (30.00) | 2.593 | 0.459 |
| **Hypertension, n (%)** | 7 (15.60) | 6 (12.00) | 1 (11.11) | 7 (14.00) | 0.309 | 0.958 |
| **Hyperlipidemia, n (%)** | 9 (20.00) | 9 (18.00) | 1 (11.11) | 11 (22.00) | 0.682 | 0.877 |
| **Diabetes mellitus, n (%)** | 5 (11.11) | 4 (8.00) | 0 (0) | 4 (8.00) | 1.270 | 0.736 |
| **Current Smoking, n (%)** | 10 (22.22) | 10 (20.00) | 2 (22.22) | 13 (26.00) | 0.525 | 0.913 |
| **Mean arterial pressure, mmHg** | 88.78±7.24 | 88.10±5.32 | 90.11±3.02 | 87.10±8.23 | 0.755 | 0.521 |
| **End-tidal CO_2_, mmHg** | 40.82±4.64 | 40.34±3.17 | 39.67±4.24 | 39.50±5.38 | 0.754 | 0.522 |

Abbreviations: PFO: patent foramen ovale.

**Table S2. Comparison of phase differences in different groups.**

|  | **PFO migraineurs**  **(n = 45)** | **Non-PFO migraineurs**  **(n = 50)** | **PFO non-migraineurs (n=9)** | **Heathy controls**  **(n = 50)** | **F** | **P** |
| --- | --- | --- | --- | --- | --- | --- |
| **Phase difference (degree)** |  |  |  |  |  |  |
| Overall | 46.25±13.02^†‡^ | 54.19±8.95 | 41.14±11.58^†‡^ | 54.46±14.28 | 4.731 | 0.003 |
| Left hemisphere | 47.45±13.49 | 54.36±9.85 | 41.16±10.85^†‡^ | 54.26±16.03 | 7.166 | <0.001 |
| Right hemisphere | 45.05±14.10^†‡^ | 54.02±9.50 | 41.13±13.93^†‡^ | 54.67±14.54 | 6.664 | <0.001 |
| **Gain** **(%/mmHg)** |  |  |  |  |  |  |
| Overall | 1.15±0.36 | 1.09±0.30 | 1.11±0.28 | 1.23±0.24 | 1.856 | 0.138 |
| Left hemisphere | 1.13±0.34 | 1.07±0.29 | 1.11±0.29 | 1.21±0.26 | 1.767 | 0.156 |
| Right hemisphere | 1.16±0.40 | 1.10±0.31 | 1.11±0.31 | 1.25±0.27 | 1.974 | 0.120 |

Abbreviations: PFO: patent foramen ovale. ^†^ P < 0.05 for comparison with non- PFO migraineurs, with post-hoc analysis performed by Bonferroni’s method; ^‡^ P < 0.05 for comparison with healthy controls, with post-hoc analysis performed by Bonferroni’s method.

**Table S3. Comparison of beat-to-beat heart rate variability parameters in different groups.**

|  | **PFO migraineurs**  **(n = 43)** | **Non-PFO migraineurs**  **(n = 49)** | **PFO non-migraineurs (n=9)** | **Controls**  **(n = 49)** | **F** | **P** |
| --- | --- | --- | --- | --- | --- | --- |
| **Time domain indices** |  |  |  |  |  |  |
| NN intervals (ms) | 864.89 ± 106.38 | 832.28 ± 140.07 | 896.61±121.44 | 834.32 ± 106.46 | 1.271 | 0.287 |
| SDNN (ms, log) | 1.58 ± 0.18 | 1.55 ± 0.18 | 1.55±0.15 | 1.61 ± 0.15 | 0.965 | 0.411 |
| RMSSD (ms, log) | 1.52 ± 0.23 | 1.48 ± 0.22 | 1.50±0.22 | 1.54 ± 0.18 | 0.680 | 0.565 |
| **Frequency domain indices** |  |  |  |  |  |  |
| TP (ms^2^, log) | 3.06 ± 0.39 | 3.02 ± 0.39 | 3.02±0.30 | 3.14 ± 0.30 | 0.968 | 0.410 |
| VLF (ms^2^, log) | 2.66 ± 0.40 | 2.59 ± 0.44 | 2.58±0.22 | 2.66 ± 0.31 | 0.395 | 0.757 |
| LF (ms^2^, log) | 2.34 ± 0.44 | 2.40 ± 0.39 | 2.33±0.37 | 2.50 ± 0.36 | 1.331 | 0.267 |
| HF (ms^2^, log) | 2.55 ± 0.49 | 2.48 ± 0.49 | 2.54±0.46 | 2.68 ± 0.40 | 1.637 | 0.183 |
| LF (nu.) | 39.71 ± 15.58 | 45.45 ± 16.18 | 39.51±17.89 | 40.66 ± 15.70 | 1.244 | 0.296 |
| HF (nu.) | 60.29 ± 15.58 | 54.55 ± 16.18 | 60.49±17.89 | 59.34 ± 15.70 | 1.244 | 0.296 |
| LF/HF | 0.93 ± 0.12 | 0.99 ± 0.19 | 0.93±0.14 | 0.94 ± 0.11 | 1.654 | 0.180 |

Abbreviations: HRV: heart rate variability; NN intervals: time interval between successive heartbeats from which artifacts have been removed; SDNN: the standard deviation of all NN intervals; RMSSD: the root mean square of successive diﬀerences of NN intervals; TP: total power. VLF, very-low-frequency. LF, low-frequency. HF, high-frequency; PFO: patent foramen ovale.
